# Room temperature bilayer water structures on a rutile TiO_2_(110) surface: hydrophobic or hydrophilic?†

**DOI:** 10.1039/d2sc02047e

**Published:** 2022-08-16

**Authors:** Mengyang Qu, Gang Huang, Xinyi Liu, Xuechuan Nie, Chonghai Qi, Huabin Wang, Jun Hu, Haiping Fang, Yi Gao, Wei-Tao Liu, Joseph S. Francisco, Chunlei Wang

**Affiliations:** Shanghai Institute of Applied Physics, Chinese Academy of Sciences Shanghai 201800 China; University of Chinese Academy of Sciences Beijing 100049 China; Department of Physics, Fudan University Shanghai 200433 China wtliu@fudan.edu.cn; Institute of Theoretical Physics, Chinese Academy of Sciences Zhongguancun East Road 55 Beijing 100190 China; Chongqing Institute of Green and Intelligent Technology, Chinese Academy of Sciences Chongqing 400714 China; Zhangjiang Lab, Interdisplinary Research Center, Shanghai Advanced Research Institute, Chinese Academy of Sciences Shanghai 201210 China wangchunlei@zjlab.org.cn gaoyi@sinap.ac.cn; School of Science, East China University of Science and Technology Shanghai 200237 China; Department of Earth and Environmental Science and Department of Chemistry, University of Pennsylvania Philadelphia Pennsylvania USA frjoseph@sas.upenn.edu

## Abstract

The lack of understanding of the molecular-scale water adsorbed on TiO_2_ surfaces under ambient conditions has become a major obstacle for solving the long-time scientific and applications issues, such as the photo-induced wetting phenomenon and designing novel advanced TiO_2_-based materials. Here, with the molecular dynamics simulation, we identified an ordered water bilayer structure with a two-dimensional hydrogen bonding network on a rutile TiO_2_(110) surface at ambient temperature, corroborated by vibrational sum-frequency generation spectroscopy. The reduced number of hydrogen bonds between the water bilayer and water droplet results in a notable water contact angle (25 ± 5°) of the pristine TiO_2_ surface. This surface hydrophobicity can be enhanced by the adsorption of the formate/acetate molecules, and diminishes with dissociated H_2_O molecules. Our new physical framework well explained the long-time controversy on the origin of the hydrophobicity/hydrophilicity of the TiO_2_ surface, thus help understanding the efficiency of TiO_2_ devices in producing electrical energy of solar cells and the photo-oxidation of organic pollutants.

## Abstract 2

An ordered water bilayer structure was identified on a rutile TiO_2_(110) surface at ambient temperature by combining VSFG experiments and MD simulations, which well explained the long-time controversy on the wetting behaviors of the TiO_2_ surface.

## Graphical abstract

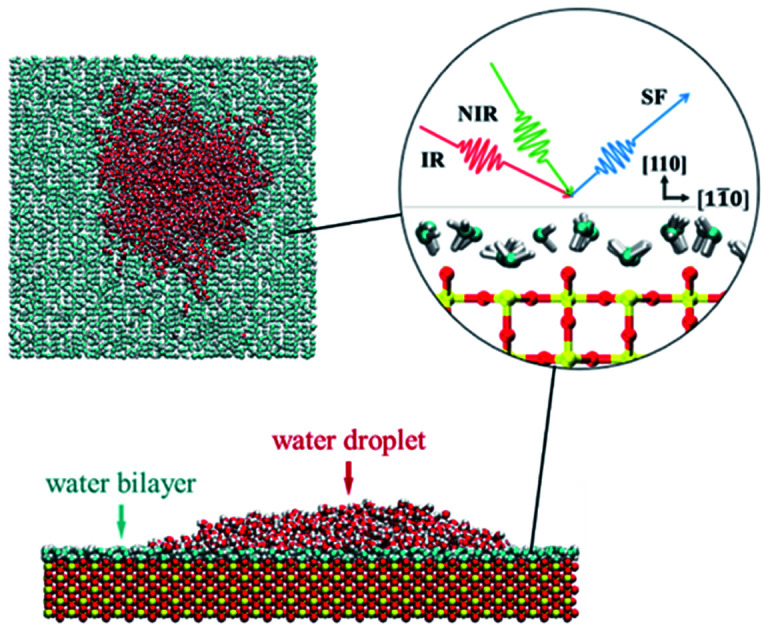

## 1 Introduction

Understanding the chemistry and physics of water on titanium dioxide (TiO_2_) is of long-time scientific interest.^1–20^ It has crucial applications in the fields of energy and environment, such as the photocatalysis for water splitting,^4–8,10,21^ self-cleaning, water purification, antifogging, heat transfer and heat dissipation,^1,2,9,11,12^ efficiency in producing electrical energy of solar cells^16,22,23^ and photo-oxidation of organic pollutants.^10^ Since the discovery of the amphiphilicity on the solid surface, the hydrophobicity/hydrophilicity of the TiO_2_ surface has been debated for decades.^1,2^ Specifically, there is still inconsistency in understanding this nonwetted phenomenon of TiO_2_ surfaces. For example, the macroscopic contact angle is around 15°–30° initially on a freshly prepared surface, but increases to around 60°–70° on the TiO_2_ surface under ambient conditions in the dark.^1,2,10,16,24–26^ However, the surface became superhydrophilic immediately after UV exposure. They attributed the nonwetted behavior to the existence of an intrinsically hydrophobic (oleophilic) region.^1,2^ The subsequent experimental studies attribute this nonwetted phenomenon to the adsorbed hydrocarbon contamination.^27,28^ In 2018, Diebold *et al.* claimed that the carboxylic acid monolayer on the rutile TiO_2_(110) surface can induce hydrophobic surfaces through the experimental method combining atomic-scale microscopy (AFM), scanning tunneling microscopy (STM) and X-ray photoelectron spectroscopy (XPS).^29^ Despite some theoretical studies that observed the molecular ordered water structure by molecular dynamics (MD) simulations,^30,31^ and experimental studies under vacuum conditions or at cryogenic temperatures,^13,14,32–35^ the intrinsic hydrophobicity/hydrophilicity of the TiO_2_ surface and its transition mechanism is still controversial due to the lack of understanding of the molecular-scale wetting mechanism. Intuitively, the strong interactions (>1.0 eV) between TiO_2_ and water^36^ should result in a superhydrophilic surface in principle exactly as on a mica surface,^37,38^ but unfortunately this is not the case. Although there have been suspicions that the hydrophobic dark state results from adventitious contamination, recent reports of the formation of a highly ordered surface structure with (2 × 1) symmetry on pristine TiO_2_ rutile (110) in H_2_O^39^ have called this into question.

Our previous studies have theoretically predicted that water droplets coexist with the ordered water layer at room temperature on model surfaces, which can be termed an unexpected phenomenon of “ordered water layer that does not completely wet water.”^40,41^ In this work, we have identified with molecular dynamics (MD) simulations an ordered water bilayer with a two-dimensional hydrogen bond (H-bond) network adsorbed on rutile TiO_2_(110) at ambient temperature, corroborated by vibrational sum-frequency generation (VSFG) spectroscopy. The computed water coverage dependence of the VSFG spectra agrees well with our measurements in the oxygen–deuterium (O–D) stretching vibration frequency range (2630 cm^−1^). These water structures together with the H-bond network result in unexpected water droplets that do not completely wet the bilayer water, which well explained the long-time controversy on the origin of the hydrophobicity/hydrophilicity of the TiO_2_ surface.

## 2 Methods

### 2.1 Laser system and the experimental setup for VSFG spectroscopy

Surface specific VSFG spectroscopy is an effective tool for probing the molecular species, configurations and bonding geometries of adsorbates on surfaces, and can work in a wide range of pressure and temperature.^15,42–46^ The incident 800 nm near-infrared (NIR) and the broadband infrared (IR) beams overlap at the sample surface to generate the sum frequency (SF) signal. The reflected spectra was collected and analyzed. The basic theory of VSFG is described in detail elsewhere.^44,45,47^ Briefly, when the IR frequency is near a surface vibrational resonance, the SF signal (*S*_SF_) is proportional to,|χNR + χR|2 where χNR is the non-resonant background, and 
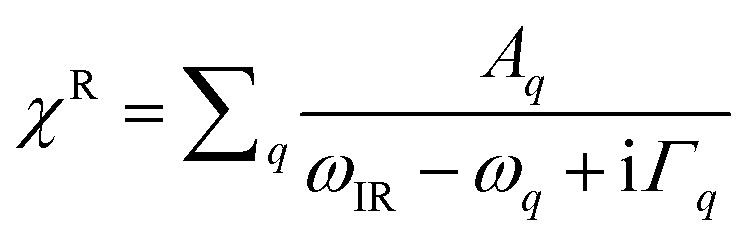
 is the resonant contribution, with *A*_q_, *ω*_q_, and *Γ*_q_ being the amplitude, frequency, and damping coefficient of the qth resonance mode, respectively.^48^ In this study, χNR is mainly from the rutile lattice, and χR from stretching vibrational modes of adsorbed water molecules. To focus on spectra from adsorbates, we set the [001] axis of the rutile (110) sample to be parallel to the beam incident plane, so that the χNR can be largely suppressed under the SSP beam polarization combination we used (S-SF, S-NIR, and P-IR).^49^

The seed light generated from a Ti : sapphire oscillator (MaiTai SP, Spectra Physics), was guided into a regenerative Ti : sapphire amplifier (Spitfire, Spectra Physics) to produce ∼4 W of 800 nm, 35 fs pulses at a 1 kHz repetition rate. The beam was divided into two parts by using a beamsplitter. About 2.6 W of the beam passed through a Bragg Filter (N013-14-A2, OptiGrate), generating narrowband pulses of 0.5 nm bandwidth. The rest passed through an optical parametric amplifier followed by a difference frequency generation stage to obtain broadband IR pulse (∼300 cm^−1^) centered at 2630 cm^−1^. The narrowband 800 nm beam of ∼12 μJ per pulse and the IR input beam, tunable from 780 to 980 cm^−1^ of ∼10 μJ per pulse, overlapped at the sample surface with incident angles of 45° and 57°, respectively. The generated SF signal was collected by using a spectrograph (Acton SP2300) and recorded on a CCD camera (Princeton Instruments PyLoN 1340 ×100).

**Fig. 1:**
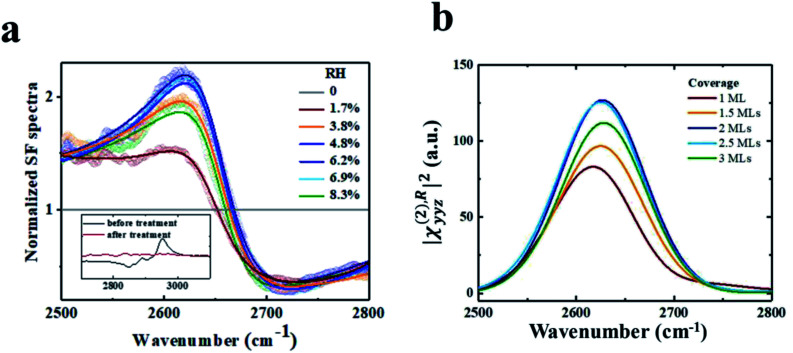
(a) Experimental VSFG spectra from D_2_O adsorbed on rutile TiO_2_(110) at varying relative humidity (RH). All spectra were recorded with an SSP combination of beam polarizations, and normalized to spectra of pristine rutile TiO_2_(110) without D_2_O. (b) Calculated SSP-VSFG spectra |*χ*^(2),R^_*yyz*_|^2^ of the interfacial D_2_O on rutile TiO_2_(110). The inset in (a) is the VSFG spectra from the rutile surface in the C–H stretch range before (black) and after (red) the UV ozone treatment. The polarization combination was SSP.

A rutile (110) crystal was bought from Hefei Kejing Materials Technology Co. Ltd. It was cleaned by sonicating in acetone (analytically pure, Shanghai Dahe chemicals Co. Ltd), ethanol (analytically pure, Shanghai Zhengxing No. 1 Chemical Plant), and deionized water (18.2 MΩ cm) for 30 minutes successively, followed by UV-ozone treatment for 30 minutes to remove organic contamination monitored using VSFG spectra. The cleanliness of the surfaces is important in this study. The inset of Fig. 1a shows the VSFG spectra of the rutile sample before (black) and after (red) UV-ozone treatment. Resonant peaks at ∼2850, 2888 and 2950 cm^−1^ are due to surface hydrocarbon contaminants. They become undetectable after cleaning. The SFG measurements were then carried out under the vacuum condition, and the signal of the contaminants remained negligible during the time period of spectral acquisition.

It is usually believed that in an ambient environment, the rutile surface is covered by water H_2_O molecules or OH moieties that are difficult to remove. To avoid the intervention of this OH background, we used deuterium oxide D_2_O in the measurement, and probed the OD stretching vibration spectra. Before the dosage of D_2_O, the pristine rutile surface exhibits no OD vibrational modes, so its spectrum can act as a good reference. During the measurement, the samples were stored in a homemade vacuum chamber with a base pressure <5 Pa. A BaF_2_ window coated with a 35 nm-thick SiO_2_ film was used to transmit input and outgoing beams, and was placed ∼3–4 mm above the sample to reduce the absorption of IR light by the 2PA vapor. D_2_O could fill the chamber to different pressures through a micro metering valve. All experiments were conducted at room temperature.

### 2.2 MD simulation methods

All MD simulations were performed with Gromacs 5.0.7 in the *NVT* ensemble (constant volume and constant temperature). The temperature was maintained at 300 K by the V-rescale method with a coupling coefficient of 0.1 ps. The particle-mesh Ewald method^50^ with a real space cutoff of 1 nm was used to treat long-range electrostatic interactions and 1 nm cutoff was applied to the van der Waals interactions. The flexible simple point charge (SPC) model^51^ was chosen for water, in which the O–H (O–D) bonds and H–O–H (D–O–D) angles are described by harmonic potentials. Of note, throughout this paper, unless specified otherwise, all simulation results were obtained from MD simulations with a flexible SPC water model. Each simulation cell for molecular dynamics contained a rutile (110) slab with a thickness of 14.4 Å composed of five TiO_2_ layers and filling the full cross section in the *x*–*y* direction, resulting in an extended contiguous surface under periodic boundary conditions. The slab structure was optimized and kept fixed during the simulation and the force field was chosen from the previous work by Bandura *et al.*^52,53^ Specifically, our model specified a H-bond between water if the O–O distance was less than 3.5 Å and simultaneously the angle H–O···O was less than 30°.^54^

For VSFG related simulations, the simulation box size is set as 65.1 × 59.2 × 200.0 Å^3^ with different numbers of D_2_O molecules covering the surfaces. A calculation of 20 ns was performed with a time step of 1 fs for equilibrium. After the 20 ns simulation, a 2 ns successive simulation was performed, while the coordinate and velocity trajectories were updated every 1 fs for SFG spectra calculation and water structure analysis. For wetting behavior simulations, the simulation box size is set as 149.76 × 147.91 × 200.0 Å^3^ with an initial rectangle water droplet containing 5000 H_2_O molecules located on the surface. The simulation time is 20 ns and the last 4 ns data were collected for analysis.

### 2.3 Methods of MD simulations with neural network potentials (NN-MD)

In recent years, MD simulations with neural network potentials (NN-MD) have been established, which combine the accuracy of first-principles methods with the efficiency of standard force fields based on machine learning. In the NN-MD simulations, the global neural network (G-NN) potential for Ti–O–H systems generated by learning the first principles dataset of global PES from stochastic surface walking (SSW) global optimization is provided by Liu’s group.^55^ Simulations were performed by LAMMPS.^56^ A simulation with a constant number of particles, constant pressure, and constant temperature (*NPT*) was performed for each system under periodic boundary conditions in all directions. A temperature of 300 K and pressure of 1 atm were maintained by the Nosé–Hoover method with a coupling coefficient of 0.1 ps. Each simulation cell contained a rutile (110) slab composed of five TiO_2_ layers, using a 4 × 2 supercell, separated by 200 Å.

For VSFG related simulations, the simulation uses one simulation cell with different numbers of D_2_O molecules covering the surfaces. A calculation of 2 ns was performed with a time step of 0.5 fs, while the coordinate and velocity trajectories were updated every 1 fs in the last 500 ps for SFG spectra calculation and water structure analysis. For wetting behavior simulations, the simulation is divided into two steps. The first step employed one simulation cell with 32 H_2_O molecules covering the surfaces. A calculation of 1 ns was performed with a time step of 0.5 fs for equilibrium. In the second step, four identical cells, from the last frame of the previous step, are stitched together along the (11̄0) crystal direction as the initial coordinates. The second-step simulation time is 5 ns and the last 3 ns data were collected for analysis.

## 3 Results and discussion

### 3.1 Ordered water bilayer adsorbed on rutile TiO_2_(110) at ambient temperature identified by VSFG experiments and MD simulation

VSFG spectroscopy is a highly sensitive technique that quantifies the surface density and orientation of adsorbates under ambient conditions. By combining VSFG experiments and MD simulations, we have identified an ordered water bilayer with a two-dimensional H-bond network adsorbed on rutile TiO_2_(110) at ambient temperature. The principle of VSFG is described in previous literature studies^43,57–61^ and in Section 2.1. Briefly, in the experiments, D_2_O was dosed onto a clean single-crystal rutile TiO_2_(110) surface in a vacuum chamber.^59^ D_2_O was used to avoid the intervention of an atmospheric O–H background. Infrared and near-infrared laser pulses were overlapped on the sample surface, which was placed with the (001) crystal direction along the incident plane to minimize the non-resonant background.^60^ The beam polarization was an SSP combination of S-polarized sum-frequency output, P-polarized near-infrared input, and P-polarized infrared input, where S denotes the polarization perpendicular to the incident plane and P denotes the polarization parallel to it.^59–61^ The vibrational spectra of the adsorbed D_2_O molecules were collected at varying relative humidity (RH). See Section 2.1 for more details about the laser system and experimental setup.

We performed MD simulations of the rutile TiO_2_(110)/water interface for various coverages, with various numbers of deuterium oxide (D_2_O) molecules in the flexible simple point charge (SPC) model^51^ covering the surface in accordance to the experiments. More details about the simulation setup can be found in Section 2.2. We recorded the VSFG spectra of the interfacial water at each coverage, focusing on the spectral characteristics of the O–D stretching frequency of water and including all water molecules in the bilayer using the obtained MD trajectories. More details about the calculation can be found in Section PS 1 of the ESI.† We note that, in the simulations, the *x* axis was set along the (001) crystal direction while the *z* axis was set perpendicular to the surface. Therefore, the SSP-VSFG response function (the resonant part of the second-order susceptibility, χ^(2),R)^_SSP_ can be classically expressed as:1
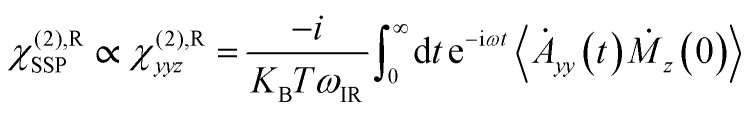
where the indices SSP refer to the polarization directions of the SFG, NIR and IR beams, respectively; *ω*_IR_ is the frequency of the IR beam; *A_yy_* and *M_z_* are respectively the components of the total polarizability tensor and the total dipole moment; the dot stands for the time derivative; and 〈…〉 stands for a statistical average.^62,63^

Fig. 1 shows the experimental spectra, showing fair accordance with the theoretical calculated VSFG spectra dependent of the D2O coverage. Both the experimental and calculated spectra exhibit a resonance mode at ∼2630 cm^−1^. The mode intensity increases with the RH increasing from ∼0% to ∼6%, corresponding to about 1.5–2 MLs of D_2_O on rutile TiO_2_(110).^64^ Interestingly, the mode intensity drops at even higher D_2_O coverage (see Fig. 1a). This trend of intensity seems can be also observed in the simulations, where the mode intensity first increases with the D_2_O coverage ranging from 0 to 2 MLs, but decreases at even higher coverage (see Fig. 1b). We also performed additional MD simulations using the flexible SPC/E water model,^65^ and MD simulations with the neural network potential (NN-MD) which combines the accuracy of first-principles methods with the efficiency of standard force fields based on machine learning. More details about the NN-MD simulations can be found in Section 2.3. The changing trend of VSFG spectrum intensity was observed in all of the simulations (see Section PS 2 in the ESI).† The peak strength of VSFG spectra not only scales with the number density of contributing moieties, but also with their orientational order.^43,57,59–61^ Therefore, both the experiment and simulation clearly show a coverage-dependent ordered structure of the interfacial water on rutile TiO_2_(110), where the orientational order of the interfacial water reaches a saturation when the coverage is approximately 2 MLs.

**Fig. 2:**
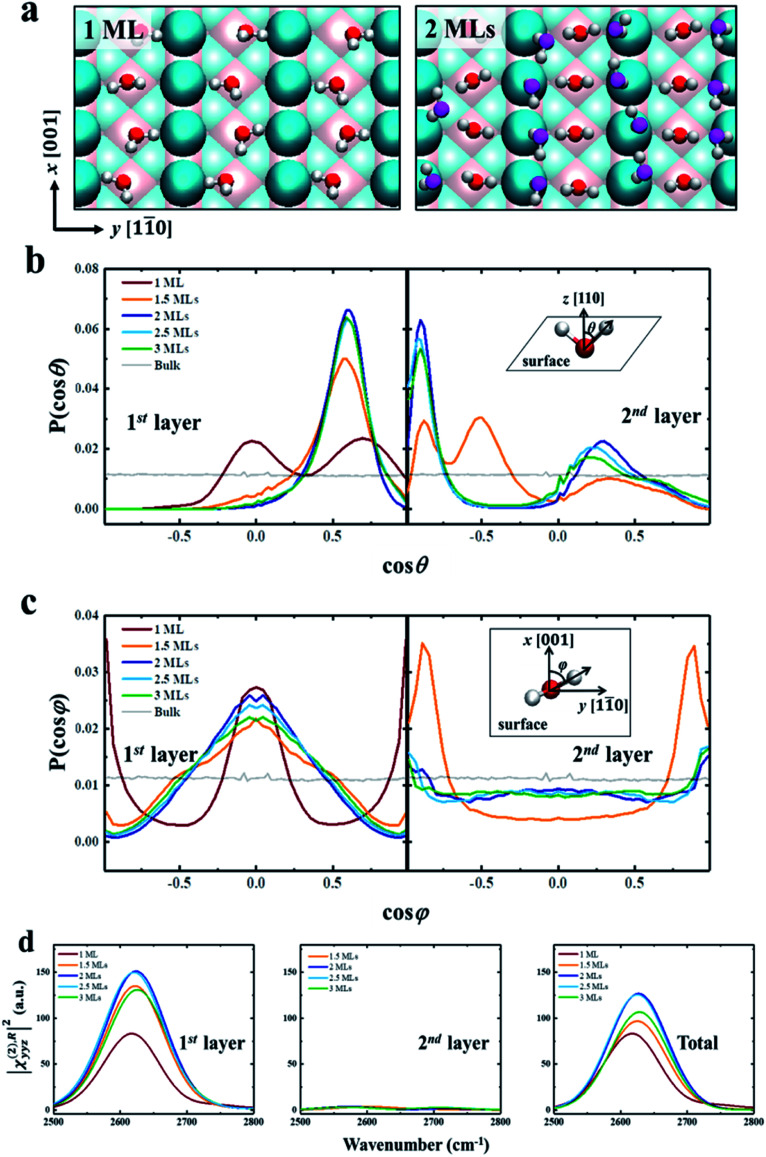
(a) Snapshots of the ordered water structures for 1 ML and 2 MLs of D_2_O adsorbed on rutile TiO_2_ (110). The atoms are color coded as follows: Ti (pink), O of TiO_2_ (cyan), O of D_2_O (first layer, red; second layer, magenta), and D (white). (b) Normalized probability distributions of the angle *θ* between the O–D group and the normal direction of the solid surface (*z* axis) for different D_2_O coverages in the first layer and second layer. (c) Normalized probability distributions of the angle *φ* between the projection of the O–D group on the surface and the (001) crystal direction (*x* axis) for different D_2_O coverages in the first layer and second layer. (d) ||*χ*^(2),R^_*yyz*_|^2^ for different D_2_O coverages for the first layer, second layer and total water. The insets of (b) and (c) are diagrams of angles *θ* and *φ*.

We further try to understand the origin of the VSFG spectra by analyzing the water molecular structures at the molecular scale. In the MD simulations, as the water coverage increases, water molecules first adsorb to the five-fold Ti sites with one molecule per Ti, and form a two-dimensional periodic monolayer (ML) with D_2_O molecules covering all of the Ti sites, as shown in the left panel of Fig. 2a. The additional water molecules adsorb on the bridge O atoms when the D_2_O coverage is over 1 ML, and forms a bilayer consisting of two ordered MLs completely covering the surface (see the right panel of Fig. 2a). This ordered water bilayer structure remains even an extra water layer added on the surface. To characterize the molecular structures of the bilayer water, we define two angle parameters *θ* and *φ* to describe the ordered water configuration in each water layer. The angle *θ* is the angle between the O–D group and the normal direction of the solid surface, and *φ* is the angle between the projection onto the surface of the O–D group and the (001) crystal direction. The normalized probability distributions of *θ* and *φ* are shown in Fig. 2b and c. For the first-layer water, the distributions of *θ* peaks at *θ* ≈ 90° (cos *θ* ≈ 0) and *θ* ≈ 45° (cos *θ* ≈ 0.71) with 1 ML coverage as shown in the left panel of Fig. 2b, which merge into one at *θ* ≈ 55° (cos *θ* ≈ 0.57) as the D_2_O coverage is larger than 1.5 ML. And the amplitude of these peaks increases to a maximum at 2 ML coverage. That is, as the coverage increases from 0 to 2 MLs, the increased deuterium bonds (D-bonds) between the first and second layers more effectively orient the first-layer O–D groups away from the surface. However, when the D_2_O coverage increases to above 2 MLs, the extra D_2_O above the bilayer forms new D-bonds with the D_2_O in the first layer and reduces the number of D-bonds between the first and second layers. This changing of D-bonding network reduces the ordered D_2_O structure of the first layer, especially in the plane parallel to the surface (see more details in PS 11), where the peaks of angle *φ*, in the left panel of Fig. 2c, significantly weakened when the coverage is greater than 2 MLs.

As shown in Fig. 2d, we have calculated the |*χ*^(2),R^_*yyz*_|^2^ in the first two layers and total water, which includes orientational ordering of interfacial water. We note that the calculated vibrational density of states (VDOS) (see Fig. S2 in the ESI†) shows that the vibrational frequency of the first layer O–D groups remains the same for different coverages. Thus the difference in amplitude of, |*χ*^(2),R^_*yyz*_|^2^ for each coverage can be attributed to the orientational polarization of the O–D transition dipole moments in the first-layer water.^66^ For the first layer, the reduced ordering of the O–D transition dipole moment reduces the amplitude of, |*χ*^(2),R^_*yyz*_|^2^, when the coverage is greater than 2 MLs, as shown in the left panel of Fig. 2d.

As for the second layer, the amplitude of, |*χ*^(2),R^_*yyz*_|^2^ in the second layer is significantly lower than it is in the first layer, as shown in the middle panel of Fig. 2d. It is because the water structures in the second layer are less ordered than in the first layer, as shown in the right panel of Fig. 2b and c. And the amplitude of |*χ*^(2),R^_*yyz*_|^2^ for the first layer is almost the same as that for the total water, as shown in the right panel of Fig. 2d. Therefore, the contribution of the second-layer water to the VSFG spectra of the interfacial water can be ignored, *i.e.*, the changing of VSFG spectra of the interface can be explained by the changing orderliness of the first-layer water with coverage, as described above. These results provide strong evidence for the existence of water bilayer structures on the rutile TiO_2_(110) surface.

### 3.2 Bilayer water structures and the surface macroscopic hydrophobicity

**Fig. 3:**
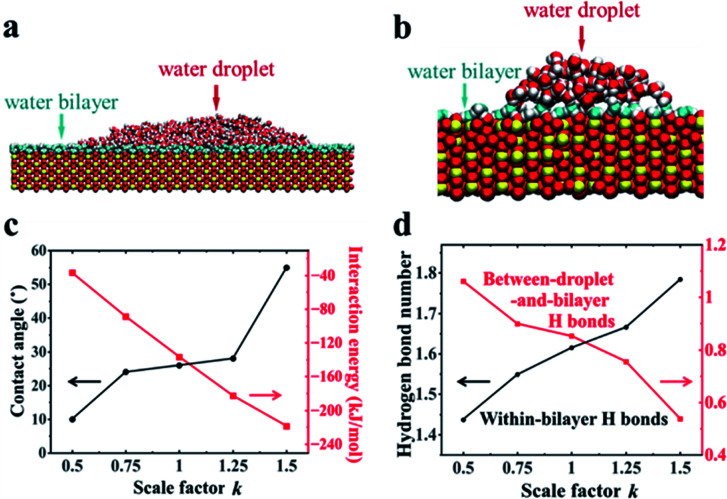
(a) Side view snapshot of the rutile TiO_2_(110) solid with a water droplet (red and white balls) coexisting with the water bilayer (cyan and white balls) in (a) MD simulations and (b) NN-MD simulations. (c) Contact angle and interaction energy per bilayer water molecule in contact with the surface *versus* scale parameter *k* of the surface atom charges, where *k* = 1 corresponds to the TiO_2_ surface in MD simulations. (d) H-bond numbers per bilayer water molecule that formed within the bilayer and between the bilayer and droplet *versus k* in MD simulations.

Further MD simulations revealed the unexpected surface wetting behavior resulting from the bilayer water structures. The simulation cell contained a rutile (110) slab composed of five TiO_2_ layers with an initial rectangle water droplet containing 5000 H_2_O molecules with a flexible SPC model^51^ located on the surfaces. Three independent systems have been tested. More details about the simulation setup can be found in Section 2.2. As shown in Fig. 3a, we have obtained a water droplet of contact angle 25 ± 5° formed on the water bilayer fully covered rutile TiO_2_(110) surface after 20 ns simulation, where the contact angle values are close to the previous experiments (32° on a freshly prepared rutile TiO_2_(110) surface).^25^ More details about the calculation of the contact angle can be found in Section PS 4 in the ESI.† We also performed the simulations using MD simulations with the other water models (see Section PS 5 in the ESI†) and NN-MD (see Fig. 3b). All of the simulations show a water droplet with a contact angle around 30°. Our results show that the freshly prepared rutile TiO_2_ surfaces can exhibit some unexpected hydrophobic behavior in pure water with quite a large contact angle even without any other contamination (such as oil). Surprisingly, as shown in Fig. 3a and b, we found that the water bilayer spreading all over the surfaces outside the water droplets. These two water bilayers, like the 2 MLs in the VSFG related simulations, have almost the same thickness of ∼0.25 nm as shown in Fig. S12.† The density profiles of the water molecules also show that there is a bilayer water structure on the surfaces (see Section PS 6 of the ESI).† Our simulation results thus show a wetting picture that a water droplet does not completely wet the water bilayer, different from the phenomenon with a water droplet on a water monolayer adsorbed on Pd(100), talc, *etc.* Of note, the wetting simulations were also performed for the rutile TiO_2_(100) surface, which also resulted in a water droplet coexisting with a water monolayer spreads all over the surface (see Section PS 7 in the ESI).† The VSFG experiments and simulations were also performed for a rutile TiO_2_(100) surface, which also shows a nonmonotonic change of the VSFG intensity with RH, same as it is on the rutile TiO_2_(110) surface, also indicating a not completely wetted ordered water monolayer on rutileTiO_2_(100).

To reveal the underlying physics of the phenomenon, we rescaled the surface charges of Ti and O by a factor of *k* (*k* = 1 corresponding to the charge of Ti and O atoms of rutile TiO_2_(110) in our simulation). As shown in Fig. 3c, we have found that the water droplet gradually spreads, and only very small contact angles (around 10°) formed on the solid surfaces when *k* = 0.5. In contrast, when *k* increased to 1.5, the water droplet formed and the contact angle increased to 55°. This clearly demonstrates the phenomenon that the more polar a surface becomes, the larger the contact angle becomes, contrary to our common sense. The results of the calculated solid–water interaction energy per bilayer water molecule in contact with the surface are shown in Fig. 3c. More details about the calculation of solid–water interaction energy can be found in Section PS 12 of the ESI.† Here we determine the thickness of the water bilayer to b *δ* = 0.30 nm for *k* = 0.5, *δ* = 0.29 nm for *k* = 0.75, *δ* = 0.27 nm for *k* = 1.0, *δ* = 0.25 nm for *k* = 1.25, and *δ* = 0.24 nm for *k* = 1.5 (see Section PS 6 in the ESI†).

We have calculated the H-bond number within the bilayer (within-bilayer H bonds) and H-bond number between the bilayer and water droplet molecules (between-droplet-and-bilayer H bonds). Fig. 3d shows the calculated number of H-bonds that each water molecule in the bilayer formed *versus* the rescale factor *k*.^40,41^ As *k* increases, more H bonds form between water molecules in the same bilayer, but fewer form between the water molecules in this layer and those above them. Thus, there is a clear competition between intra-bilayer H bonds and H bonds between the droplet and bilayer. The decreasing number of H-bonds within the bilayer and those above it causes the weaker interaction between the bilayer and the droplet.^40,41^ This is consistent with the unexpected increase in the contact angle of the droplet as *k* increases. For the rutile TiO_2_(110) surfaces (*k* = 1.0), the intra-bilayer H bonds is 1.6 per water molecule, which is almost twice that of 0.85 per water molecule between the droplet and bilayer, showing that the water in the bilayer prefers to form H-bonds within the bilayer. The analyses above clearly show that the bilayer water structures with the H-bonding network play a central role in understanding the surface wetting behaviors. The stronger the solid surface–water interactions are, the more stable the water bilayer structures become. This stable water bilayer further decreases the H bond number between the bilayer water and the water droplet, thus makes the bilayer more hydrophobic and enhances the contact angles of the water droplets as *k* increases.

### 3.3 Hydrophobicity/hydrophilicity of the TiO_2_ surface

**Fig. 4:**
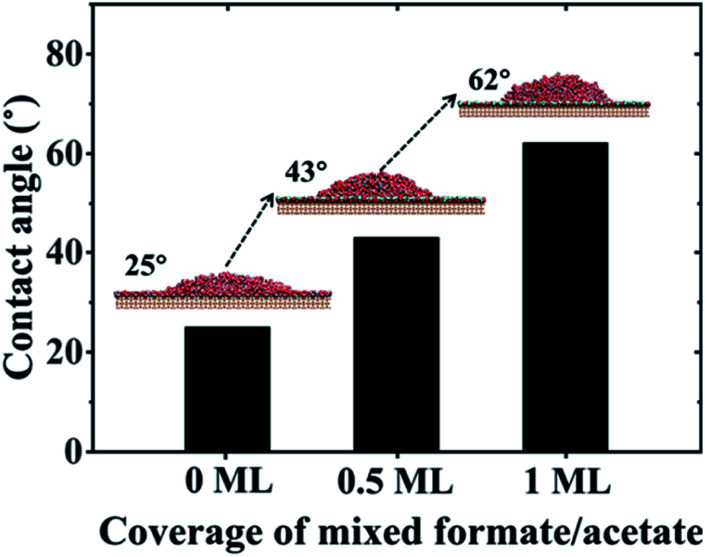
Contact angle values of water droplets on rutile TiO_2_(110) surfaces *versus* coverage of mixed formate/acetate adsorbed on the surfaces.

Diebold *et al.* found the spontaneous formation of mixed formate/acetate monolayers by adsorption from the atmosphere on the rutile TiO_2_(110) surface through the experimental method combining atomic-scale microscopy and spectroscopy.^29^ Here, we performed the process of this spontaneous adsorption of formate/acetate molecules on the molecular scale by MD simulations. More details can be found in Section PS 8 of the ESI.† Our results clearly show that the initial water bilayer adsorbed on the surface will gradually be replaced by the mixed formate/acetate layer, similar to the experiments in the dark. The presence of this mixed formate/acetate layer significantly increased the surface hydrophobicity. We have performed MD simulations of rutile TiO_2_(110) surfaces with different coverages of 1 : 1 mixed formate/acetate layers adsorbed on the surfaces (see simulation details in Section PS 9 of the ESI).† And when the coverage reaches 0.5 ML and 1 ML, the contact angles of water droplets on the surfaces increase to 43° and 62°, respectively, as shown in Fig. 4, consistent with the previous experiments where the contact angles increase from 32° to 61° in the dark.^25^ It has been observed that the surface became superhydrophilic after UV exposure with the reduction of the amount of adsorbed molecular water and the enhancement in the amount of adsorbed dissociated water with the –OH at the surface.^1,2,67^ Meanwhile, UV irradiation may also induce the photodecomposition of formic/acetic acid on the rutile TiO_2_(110) surface.^68,69^ Thus, we have performed the wetting behavior simulation of the rutile TiO_2_(110) surface with 5% and 10% covering ratios of –OH groups, by randomly planting the –OH groups on the solid surfaces. The contact angle of the water droplet on the water bilayer decreased to 19° at a covering ratio of 5% and disappeared at a covering ratio of 10%, as shown in Fig. S10.† This can be attributed to the disruption of the water bilayer hydrogen network, which transforms the hydrophobicity of the water bilayer to superhydrophilic (see details in Section PS 10 of the ESI†).

## 4 Conclusions

We combined VSFG experiments and MD simulations to identify an ordered water bilayer structure on a rutile TiO_2_ surface under ambient conditions for the first time. The ordered water structure reduces the H-bonds between the bilayer and the water molecules above it, and thus results in a notable contact angle on the water bilayer on the solid surface. The adsorption of the formate/acetate molecules and dissociated H_2_O molecules greatly affect the wetting behaviors. This work provides evidence of molecular-scale hydrophilicity^70^ and highlights the importance of a microscopic water molecular structure in understanding macroscopic behaviors.^71–74^ These results help understand the long-time conflict on the hydrophobicity/hydrophilicity of the TiO_2_ surface in previous photo-induced super-hydrophilicity of water on a TiO_2_ surface, which can be attributed to the disruption of the ordered water bilayer. We believe that this work can make a step in understanding TiO_2_-based devices in applications of self-cleaning surfaces, the electrical energy of solar cells and the photo-oxidation of organic pollutants.

## Data availability

Data available on request.

## Author contributions

C. W., Y. G., W. L. and J. F. designed the research; M. Q., G. H., X. L., C. W. and X. N. performed the research; M. Q., G. H., X. N., X. L. and C. Q. contributed new analytic tools; M. Q., G. H., X. N., X. L., H. W., J. H., H. F., Y. G., W. L. and C. W. analysed the data; M. Q., G. H., Y. L., Y. G., W. L., J. F. and C. W. wrote the paper.

## Conflicts of interest

The authors declare no conflict of interest.

## Supplementary Material

SC-013-D2SC02047E-s001
