# Supplementary material for: Room temperature bilayer water structures on a rutile TiO2(110) surface: hydrophobic or hydrophilic?
Source: Chem Sci. 2022 Aug 16;13(35):10546–54. doi: 10.1039/d2sc02047e (PMC9473646; doi:10.1039/d2sc02047e)
Supplement: SC-013-D2SC02047E-s001 [file SC-013-D2SC02047E-s001.pdf]

Electronic Supplementary Material (ESI) for Chemical Science.
This journal is © The Royal Society of Chemistry 2022

Supporting Information for

Room Temperature Bilayer Water Structures on Rutile $TiO_2(110)$ Surface: Hydrophobic or Hydrophilic?

Mengyang Qu§[a,b], Gang Huang§[d], Xinyi Liu§[c], Xuechuan Nie[a,b], Chonghai Qi[a], Huabin Wang[e], Jun Hu[a,f], Haiping Fang[g], Yi Gao[a,f]*, Wei-Tao Liu[c]*, Joseph S. Francisco[h]* and Chunlei Wang [a,f]*

[a.] *Shanghai Institute of Applied Physics, Chinese Academy of Sciences, Shanghai 201800, China.*
[b.] *University of Chinese Academy of Sciences, Beijing, 100049, China*
[c.] *Department of Physics, Fudan University, Shanghai 200433, China Email: wtliu@fudan.edu.cn*
[d.] *Institute of Theoretical Physics, Chinese Academy of Sciences, Zhongguancun East Road 55, 100190 Beijing, China*
[e.] *Chongqing Institute of Green and Intelligent Technology, Chinese Academy of Sciences, Chongqing, 400714, China*
[f.] *Zhangjiang Lab, Interdisplinary Research Center, Shanghai Advanced Research Institute, Chinese Academy of Sciences, Shanghai, China 201210 E-mail: wangchunlei@zjlab.org.cn, gaoyi@sinap.ac.cn*
[g.] *School of Science, East China University of Science and Technology, Shanghai 200237, China*
[h.] *Department of Earth and Environmental Science and Department of Chemistry, University of Pennsylvania, Philadelphia, PA, United States Email: frjoseph@sas.upenn.edu*
§ These authors contributed equally

PS 1. Calculation of the vibrational sum-frequency generation (VSFG) spectroscopy

In the VSFG simulations, the x-axis was set along the (001) crystal direction while the z-axis was set perpendicular to the surface. Therefore, the SSP-VSFG response function (the resonant part of the second-order susceptibility, $\chi^{(2),R}_{SSP}$) can be classically expressed as

$$\chi^{(2),R}_{SSP} \propto \chi^{(2),R}_{yyz} = \frac{-i}{k_B T \omega_{IR}} \int_0^{\infty} dt e^{-i\omega t} \langle \dot{A}_{yy}(t) \dot{M}_z(0) \rangle \quad , \qquad (1)$$

where ω_{IR} is the frequency of the IR beam; A_{yy} and M_z are respectively the components of the total polarizability tensor and dipole moment; the dot stands for the time derivative; and $\langle\cdots\rangle$ stands for a statistical average. A computationally efficient algorithm based on the velocity-velocity correlation function was used to obtain the time-correlation function of the dipole moment and polarizability. In this method,[1, 2] the total polarizability and dipole moment are first decomposed into their molecular components,

$$\begin{cases} \dot{M}_z = \sum_i \dot{\mu}_{z,i} \\ \dot{A}_{yy} = \sum_i \dot{\alpha}_{yy,i} \end{cases}, \tag{2}$$

where $\dot{\mu}_i$ and $\dot{\alpha}_i$ are the time derivatives of the diploe moment and polarizability of O-D bond with index i. They are then expressed with respect to the time derivative of the O-D bond length r_i,

$$\begin{cases} \dot{\mu}_{z,i} = \left[D_i(t)\left(\frac{\partial \mu^b}{\partial r}\dot{r}_i\right)\right]_z \\ \dot{\alpha}_{yy,i} = \left[D_i(t)\left(\frac{\partial \alpha^b}{\partial r}\dot{r}_i\right)D_i^T(t)\right]_{yy} \end{cases}, \tag{3}$$

where b refers to the bond framework, and $D_i(t)$ is the rotation matrix from bond to the lab framework. And the average values for the bond length derivatives of dipole and polarizability were calculated thanks to the Maximally Localized Wannier Functions (MLWF) in a bulk of D_2O.[1, 3] We finally got the simplified $\chi^{(2,R)}_{yyz}$ by substituting Eq. 2 and Eq. 3 to Eq. 1 and neglecting the inter-molecular couplings.

PS 2. The VSFG calculation results of the rutile TiO_2 (110) surface using MD simulations with the flexible extended simple point charge (SPC/E) water model and MD simulations with neural network (NN) potential

We have also performed VSFG simulations of the rutile TiO_2(110)/water interface

using a flexible SPC/E water model[4] and NN potential to confirm the robustness of the coverage-dependent ordered water bilayer structure. For each set of the simulation, the mode intensity first increases with the D_2O coverage ranging from 0 to 2 MLs but decreases at even higher coverage (see Fig. S1), which is quite consistent with the simulation results with the flexible SPC water model (Fig 1b in main text).

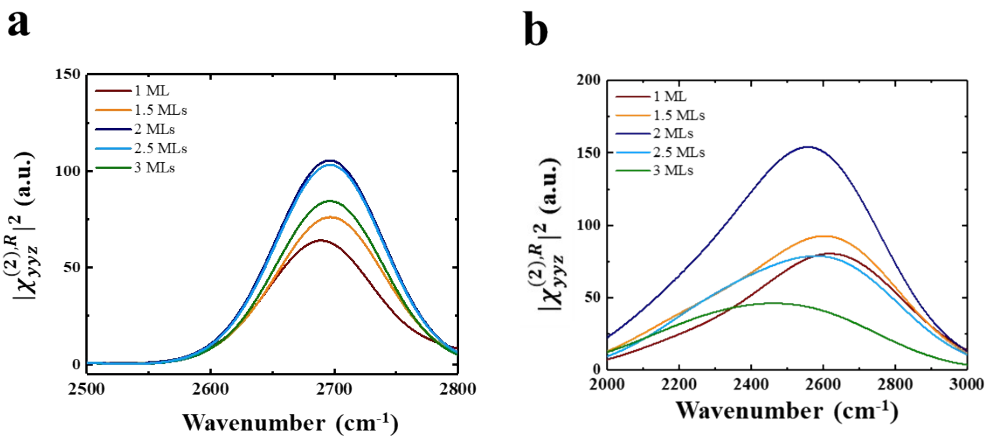

Fig. S1. Calculated VSFG spectra of the interfacial D_2O on rutile TiO_2(110) using (a) flexible SPC/E water model and (b) NN-MD.

PS 3. Vibrational density of states (VDOS) from D_2O adsorbed on rutile TiO_2 (110) for the first layer

We have calculated the vibrational density of states (VDOS) [5] from the D_2O adsorbed on the rutile TiO_2 (110) surface in MD simulations with the flexible SPC water model for the first layer. The calculated VDOS for different D_2O coverages is shown in Fig. S2. There are two peaks at ~2600 cm^{-1} and ~2700 cm^{-1} for each coverage indicating two vibration states of the -OD group, i.e., ~2600 cm^{-1} for the weak deuterium bonded -OD and ~2700 cm^{-1} for the free -OD. The nearly constant vibration frequency for different D_2O coverages indicates that the difference in VSFG spectral intensity can mainly be attributed to the orientational polarization effect on the interfacial water -OD transition dipole moments.

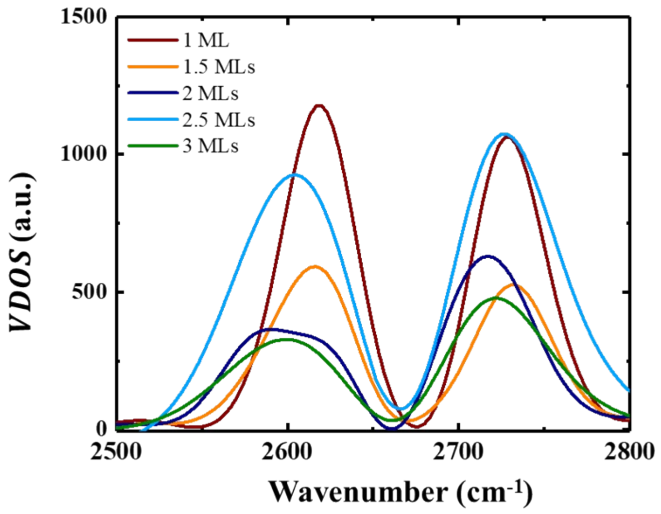

Fig. S2. Calculations of VDOS from first-layer D_2O adsorbed on the rutile TiO_2 (110) for different D_2O coverages.

PS 4. Calculation of contact angles of water droplets

To obtain a contact angle, the density distribution profile of the droplet is firstly calculated. The regions with half bulk water density (0.45-0.55 g/cm3) are defined as the edge of the droplet. The edge is then fitted with a circular shape. The contact angles are measured from where the fitted circular arcs meet the surfaces. For case that the contact angles may vary in different directions, in the MD simulations the droplets are irregular hemispheres, thus it is easy to calculate contact angles for different directions. And we determined the contact angle as the average of the contact angles in two directions of $[001]$ and $[1\bar{1}0]$. As for the NN-MD simulations, the droplets are in cylindrical, thus we have simulated the cylindrical droplets both in directions of $[001]$ and $[1\bar{1}0]$. Then we determined the contact angle as the average of the contact angles in the two directions. Three independent systems have been run to get the error bar. The converged contact angle values vs. the simulation time are shown in Fig. S3.

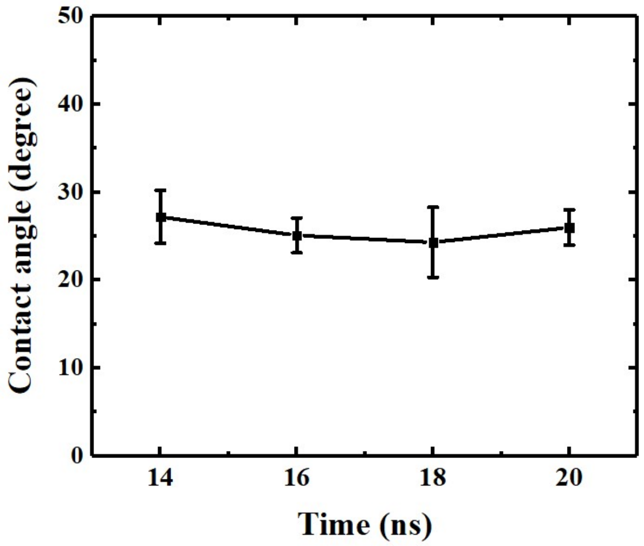

Fig. S3. Average contact angle with error bars of the droplets versus simulation time in the MD simulation.

PS 5. Additional simulations of the wetting behavior of the rutile TiO_2 (110) surface

we have performed several additional simulations including (i) classical MD simulations with different water models, and (ii) MD simulations with neural network potentials based on DFT calculations.

For the (i) classical MD simulations with different water models, additional simulations with SPC, SCP/E, Tip4p, Tip4p/ice and Tip4p/2005 water models have been performed. For each water model, an initial rectangular water droplet was located on the surfaces, after 20 ns simulation, all of the systems obtained a water droplet with a contact angle close to the previous experiments (32° on a freshly prepared rutile TiO_2(110) surface) formed on a water bilayer fully covered rutile TiO_2(110) surface, as shown in Fig. S4.

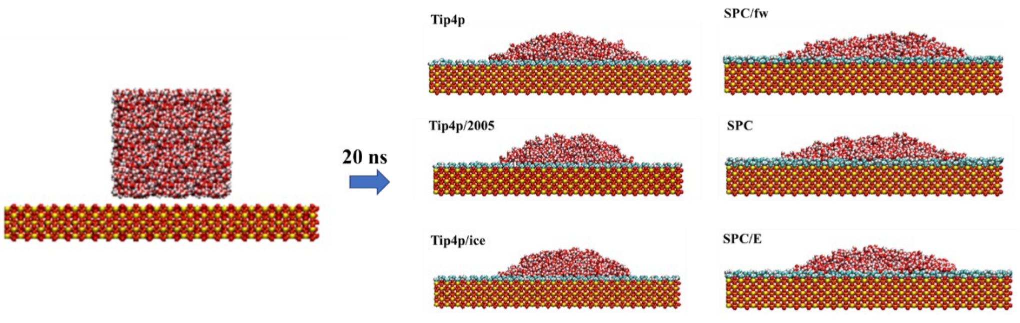

Fig. S4. Side view snapshots of initial and final conformations of wetting simulations for classical MD simulations.

For the (ii) MD simulations with neural network potentials based on DFT calculations, the first step employed one simulation cell composed of five TiO_2 layers, using a 4 × 2 supercell, covered with 32 H_2O molecules. A calculation of 1 ns was performed for equilibrium. In the second step, four identical cells, from the last frame of the previous step, are stitched together along the $(1\bar{1}0)$ crystal direction as the initial coordinates. After 5 ns simulation, the cylindrical water droplet with a contact angle of 30±5 ° coexisting with the water bilayer was also obtained as shown in Fig. S5. Note that neural network potentials combine the accuracy of first-principles methods with the efficiency of standard force fields. In the NN-MD simulations, the global neural network (G-NN) potential for Ti-O-H systems generated by learning the first principles dataset of global PES from stochastic surface walking (SSW) global optimization is provided by Liu group.[6] Simulations were performed by LAMMPS[7] and were divided into two steps.

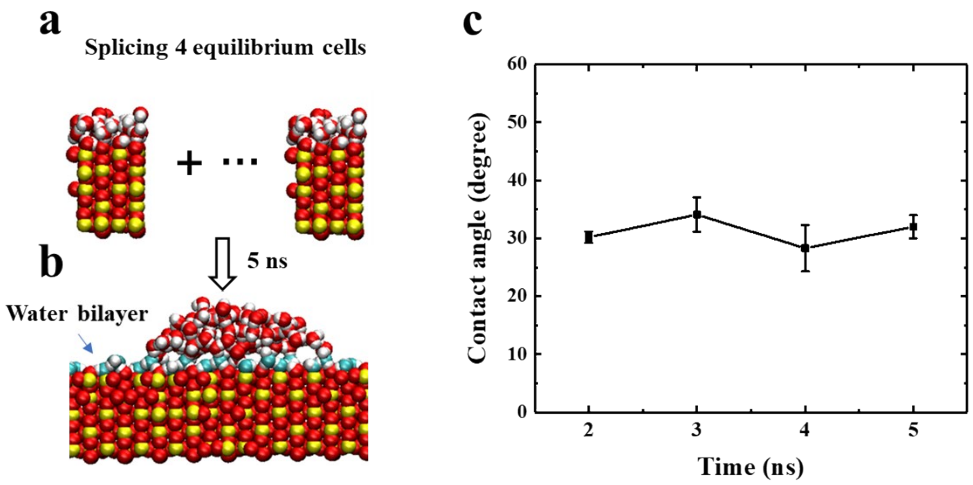

Fig. S5. (a) Side view snapshot of the simulation cell in the first-step wetting behavior NN-MD simulation. (b) Side view snapshot of rutile TiO_2(110) solid with a water droplet (red and white balls) coexisting with the water bilayer (cyan and white balls) obtained in the second-step simulation. (c) Average contact angle of the droplets versus simulation time in the second-step simulation.

All of the simulations support the main idea of our work that the ordered water bilayer structure results in an unexpected water droplet that does not completely wet the bilayer water.

PS 6. The density profile of the water molecules near the rutile TiO_2(110) surfaces in the z-direction

We have investigated the distribution of the water molecules near the TiO_2 surfaces for different scale parameters *k* of the surface charge, where the density profile of the surface water is shown in Fig S6. We determine the thickness of the water bilayer for different scale parameters *k* based on the location of the second valley of the density profiles in Fig. S6, where the thickness δ = 0.30 nm for *k* = 0.5, δ = 0.29 nm for *k* = 0.75, δ = 0.27 nm for *k* = 1.0, δ = 0.25 nm for *k* = 1.25 and δ = 0.24 nm for *k* = 1.5.

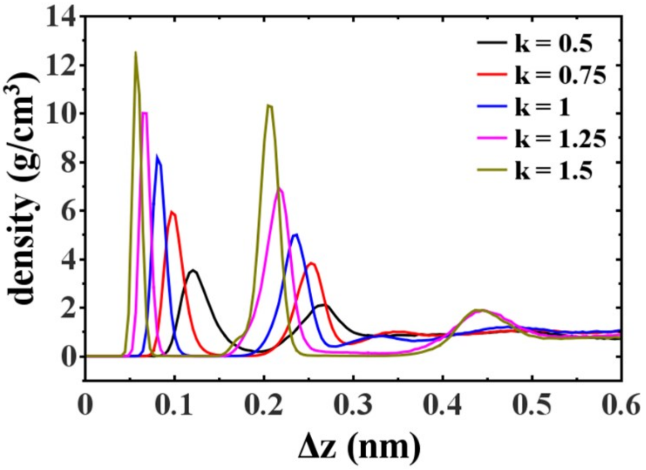

Fig. S6. Water density distribution near the rutile TiO_2(110) surfaces for different scale parameters k of the surface charge, where Δz means the vertical distance on the z-axis to the surface bridging oxygen atoms.

PS 7. Water droplets on the water monolayer on the rutile TiO_2(100)

We have also performed wetting simulations for the rutile TiO_2(100) surface using NN-MD, which also resulted in a water droplet coexisting with a water monolayer

spreading all over the surface as shown in Fig. S7.

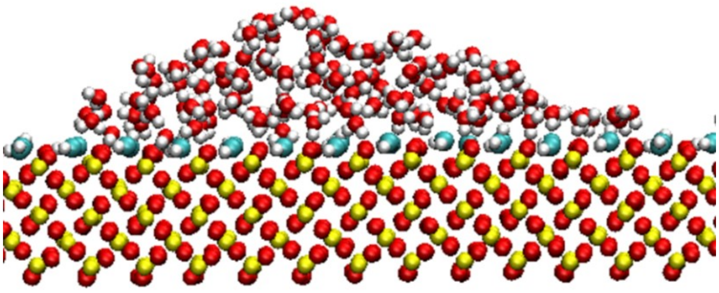

Fig. S7. Side view snapshot of rutile TiO_2(100) solid with a water droplet (red and white balls) coexisting with the water monolayer (cyan and white balls) based on the NN-MD simulation

The VSFG experiments and simulations were also performed for a rutile TiO_2(100) surface. Fig. S8 displayed the experimental and theoretical calculated VSFG spectra dependent on the D_2O coverage. In the experiment, the mode intensity increases with the relative humidity (RH) increasing from ~0% to ~30% but drops at even higher D_2O coverage (see Fig. S8a). The nonmonotonic change of the intensity with D_2O coverage, same as the previous results on the rutile TiO_2(110) surface, was also observed in the theoretical calculation where the mode intensity first increases with the D_2O coverage ranging from 0 to 1 ML but decreases at even higher coverage (see Fig. S8b). The experiment and simulation show an ordered structure of the interfacial water on rutile TiO_2(100), consistent with our previous measurements on the rutile TiO_2(110) surface.

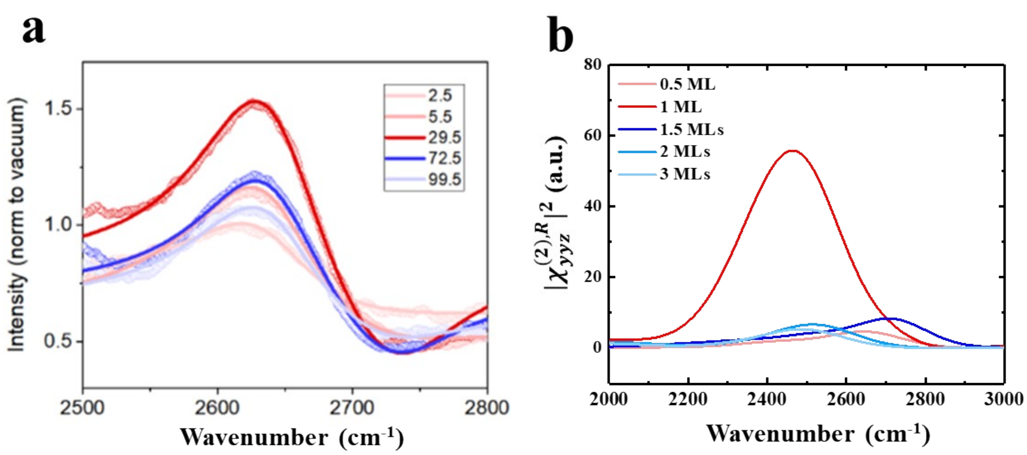

Fig. S8. (a) Experimental VSFG spectra from D_2O adsorbed on rutile TiO_2(100)

at varying relative humidity (RH). (b) Calculated SSP-VSFG spectra $\left|\chi^{(2),R}_{yyz}\right|^2$ of the interfacial D_2O on rutile TiO_2(100) at varying D2O coverage using NN-MD.

PS 8. Simulation of the adsorption process of atmospheric carboxylic acids on a rutile TiO_2(110) surface

We have performed MD simulations of the adsorption process of atmospheric carboxylic acids on a rutile TiO_2(110) surface at the molecular scale. The force field parameters of TiO_2-organic acid system are from the tools by Mark et al. [8]cited over 900 times. The force field is derived from a multistep process from quantum calculations. We firstly deposited a water bilayer on the surface. After 4 ns equilibrium, the aqueous solution of mixed formic/acetic acid molecules with a concentration of ~117 g/L was deposited on the water bilayer. The mixed formic/acetic acid molecules began to replace the position of the water molecules in the bilayer with their hydroxyl "heads". As shown in Fig. S9, the mixed formic/acetic acid molecules spontaneously replace ~28.7 water molecules in the bilayer, on average, every 4 ns. These results clearly show that the initial water bilayer adsorbed on the surface will gradually be replaced by the mixed formate/acetate monolayer in the dark.

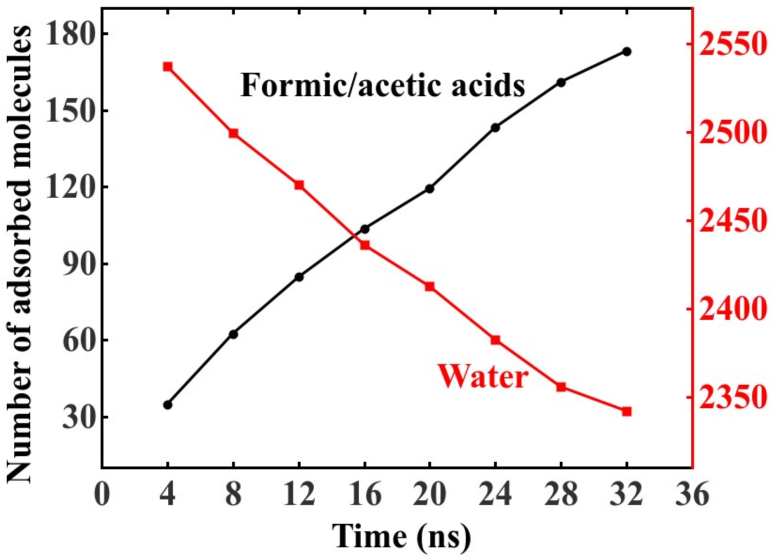

Fig. S9. The number of adsorbed molecules for formic/acetic acids and water

versus simulation time.

PS 9. Wetting behavior simulation of rutile TiO_2(110) surface with adsorbate of carboxylic acids.

We have taken out the mixed formate/acetate layer adsorbed on the rutile TiO_2(110) surface by MD simulation. The mixed formic/acetic acid molecules were firstly dissociated and the dissociated H^+ was bonded with the bridge O atoms using harmonic potential. A mixed formate/acetate layer with different coverages was then covered on the surface. After a simulation of 8 ns, all of the formates/acetates were orientated with their CHx-terminated "tails" pointed away from the surface. These CHx-terminated "tails" cause the increase in surface hydrophobicity. When we deposited an initial rectangle water droplet containing 5000 H_2O molecules on the mixed formate/acetate layer with coverages of 0.5 ML and 1 ML, the contact angles of the water droplet increased to 43° and 62°, after 20 ns simulation, respectively.

PS 10. Simulation of the wetting behavior of rutile TiO_2(110) surface with the existence of -OH groups on the surface

We have performed the wetting behavior simulation of rutile TiO_2(110) surface with 5% and 10% covering ratios of -OH groups, by randomly planting the -OH groups on the solid surfaces. The contact angle of the water droplet on the water bilayer decreased to 19° at a covering ratio of 5% and disappeared at a covering ratio of 10%, as shown in Fig. S10. We have also calculated the solid-water interaction energies per water molecule, the absolute value interaction energy decreases from 137 kJ/mol to 87kJ/mol as the increase of the ratio. Despite of the strong interaction, the hydrogen bond number between the bilayer and water droplet molecules is more important for this wetting transformation. We have calculated the hydrogen bond numbers per bilayer water molecule that formed between bilayer and droplet for different covering ratios of -OH groups. As the covering ratio increases to 5% and 10%, the hydrogen bond number per bilayer water molecule that formed between bilayer and droplet increases from 0.85 to 0.95 and 1.18 respectively. The increasing hydrogen bond number between bilayer

and droplet indicates that the water bilayer hydrogen network is gradually disrupted.

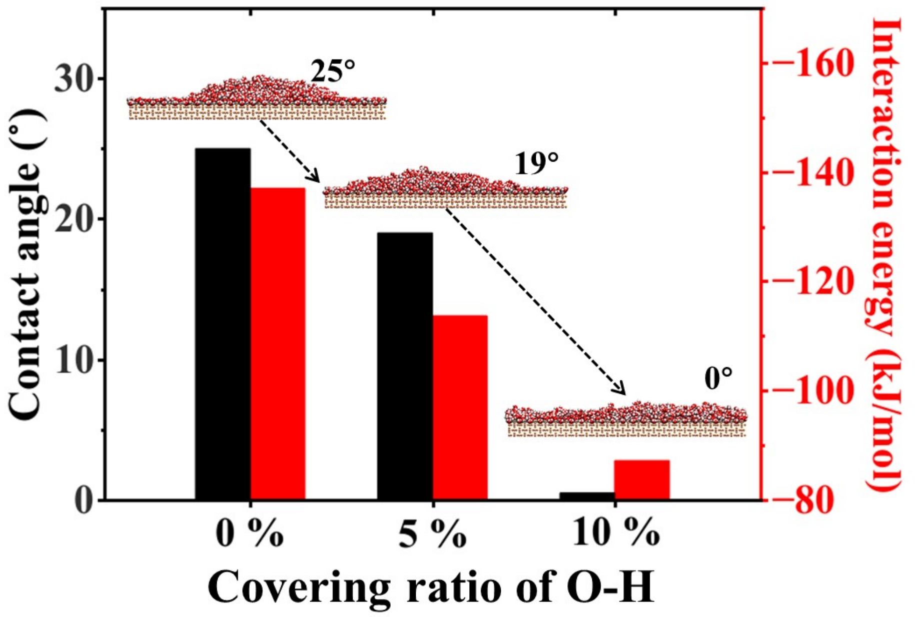

Fig. S10. Contact angle values of water droplets and the solid-water interaction energies per water molecule in the water bilayer versus the covering ratio of -OH groups on rutile TiO_2(110) surfaces.

PS 11. Disruption of the orderliness of the first-layer water when the D_2O coverage increases above 2 MLs on rutile TiO_2(110) surfaces

The D-bond network of the water bilayer is shown in Fig. S11. As we can see, the orientation of D bonds between the first and second layers is mainly along the $(1\bar{1}0)$ crystal direction, thus increase the orderliness along the $(1\bar{1}0)$ crystal direction of D_2O in the first layer. When the D_2O coverage increases above 2 MLs, the extra D_2O above the bilayer forms new deuterium bonds with the D_2O in the first layer and reduces the number of deuterium bonds between the first and second layers, thus reducing the orderliness along the $(1\bar{1}0)$ crystal direction of D_2O in the first layer.

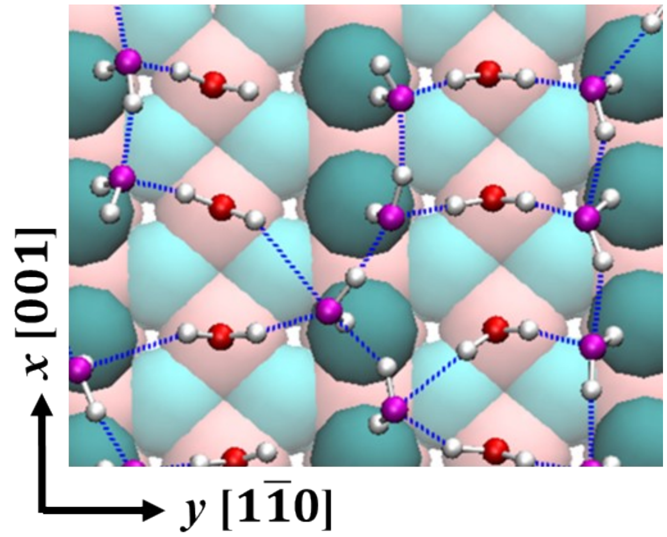

Fig. S11. D-bond network of the water bilayer on rutile TiO_2(110).

PS 12. Calculation of the solid-water interaction energy

The interaction energy between two atoms *i, j* is described as, $E_{ij} = E_{ij}^{vdw} + E_{ij}^{Coul}$, where E_{ij}^{vdw} stands the van der Waals interaction and E_{ij}^{Coul} stands the Coulomb interaction. For the van der Waals interaction, we use the Buckingham potential in the simulations, which is described as,

$$E_{ij}^{vdw} = A_{ij}\exp\left(-B_{ij}r_{ij}\right) - \frac{C_{ij}}{r_{ij}^{6}} \quad (1),$$

where, i and j stand different atoms, r_{ij}means the distance between the atoms, A_{ij}, B_{ij} and C_{ij} are Buckingham parameters between the atoms. The Coulomb interaction is described as,

$$E_{ij}^{Coul} = \frac{q_i q_j}{4\pi\epsilon r_{ij}} \quad (2),$$

where q_i and q_j stand the charges of atom *i* and *j* respectively. The solid-water interaction energy is calculated with the average interaction energy per bilayer water molecule in contact with the surface.

PS 13. Thickness comparison between water bilayers obtained from MD and NN-MD

We have investigated the distribution of the water molecules near the rutile (110) TiO_2 surfaces for both MD and NN-MD simulations, as shown in Fig. S12. We determine the thickness of the water bilayer based on the location of the second valley of the density profiles. As we can see, both of the thicknesses of the two bilayers are ~0.25 nm.

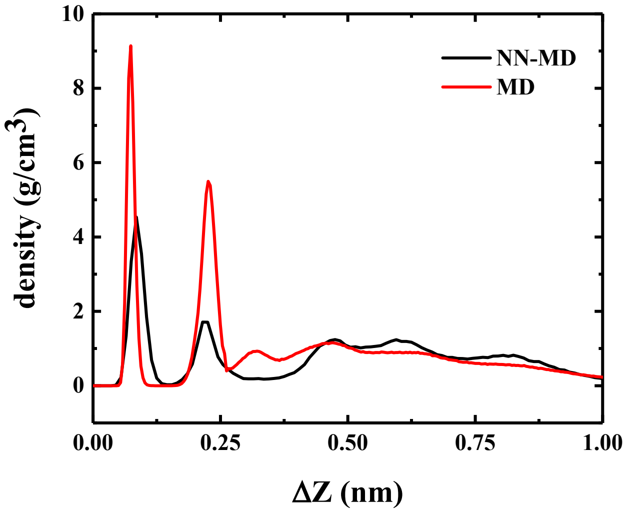

Fig. S12. Water density distribution near the rutile TiO_2(110) surfaces in NN-MD and MD simulations, where ΔZ means the vertical distance on the z-axis to the surface bridging oxygen atoms.

Reference

1. R. Khatib and M. Sulpizi, *J. Phys. Chem. Lett.*, 2017, **8**, 1310-1314.
2. R. Khatib, E. H. Backus, M. Bonn, M. J. Perez-Haro, M. P. Gaigeot and M. Sulpizi, *Sci. Rep.*, 2016, **6**, 24287.
3. N. Marzari and D. Vanderbilt, *Phys. Rev. B*, 1997, **56**, 12847.
4. P. K. Yuet and D. Blankschtein, *J. Phys. Chem. B*, 2010, **114**, 13786-13795.
5. M. Thomas, M. Brehm, R. Fligg, P. Vohringer and B. Kirchner, *Phys. Chem. Chem. Phys.*, 2013, **15**, 6608-6622.
6. S. D. Huang, C. Shang, P. L. Kang, X. J. Zhang and Z. P. Liu, *Wiley Interdiscip. Rev. Comput. Mol. Sci.*, 2019, **9**, e1415.
7. A. P. Thompson, H. M. Aktulga, R. Berger, D. S. Bolintineanu, W. M. Brown, P. S. Crozier, P. J. in 't Veld, A. Kohlmeyer, S. G. Moore, T. D. Nguyen, R. Shan, M. J. Stevens, J. Tranchida, C. Trott and S. J. Plimpton, Comput. Phys. Commun., 2022, **271**, 108171.
8. A. K. Malde, L. Zuo, M. Breeze, M. Stroet, D. Poger, P. C. Nair, C. Oostenbrink and A. E. Mark, *J. Chem. Theory Comput.*, 2011, **7**, 4026-4037.

[a] Shanghai Institute of Applied Physics, Chinese Academy of Sciences, Shanghai 201800, China.

[b] University of Chinese Academy of Sciences, Beijing, 100049, China

[c] Department of Physics, Fudan University, Shanghai 200433, China Email: wtliu@fudan.edu.cn

[d] Institute of Theoretical Physics, Chinese Academy of Sciences, Zhongguancun East Road 55, 100190 Beijing, China

[e] Chongqing Institute of Green and Intelligent Technology, Chinese Academy of Sciences, Chongqing, 400714, China

[f] Zhangjiang Lab, Interdisplinary Research Center, Shanghai Advanced Research Institute, Chinese Academy of Sciences, Shanghai, China 201210 E-mail: wangchunlei@zjlab.org.cn, gaoyi@sinap.ac.cn

[g] School of Science, East China University of Science and Technology, Shanghai 200237, China

[h] Department of Earth and Environmental Science and Department of Chemistry, University of Pennsylvania, Philadelphia, PA, United States Email: frjoseph@sas.upenn.edu

§ These authors contributed equally

Electronic Supplementary Information (ESI) available: [details of any supplementary information available should be included here]. See DOI: 10.1039/x0xx00000x
